# Lactate level, etiology, and mortality of adult patients in an emergency department: a cohort study

**DOI:** 10.1186/1757-7241-23-S1-A33

**Published:** 2015-07-16

**Authors:** Mathilde Pedersen, Vibeke Brandt, Jon G Holler, Annmarie T Lassen

**Affiliations:** 1 Department of Emergency Medicine, OUH Odense University Hospital, Odense, Denmark

## Background

Increased lactate is associated with high mortality among patients in the emergency department (ED) with suspected infection or trauma, but the association to patients with other etiologies is less well described. The aim of this study was to describe the relation between lactate, etiology, and 7-day mortality in adult ED patients.

## Methods

A retrospective cohort study of all adult patients who had lactate measured within 4 hours after arrival to the ED at Odense University Hospital between June 2012 and May 2013. The categorization of suspected etiology was based on discharge diagnoses.

## Results

5,360 patients were included. 51.7% were male, and the median age was 67 years (IQR 50-79). 77.2% had low lactate (0-1.9 mmol/L), 16.2% intermediate lactate (2-3.9 mmol/L), and 6.6% high lactate (≥4 mmol/L). 7-day mortality was 2.9% (95% CI 2.4-3.5%) for patients with low lactate, 7.8% (95% CI 6.1-9.8%) for patients with intermediate lactate, and 23.9% (95% CI 19.6-28.8%) for patients with high lactate. There was a significant trend for increasing 7-day mortality with increasing lactate among patients with a discharge diagnosis categorized as infectious (N = 1,133), cardiologic (N = 357), respiratory (N = 633), hypovolemic (N = 205), or gastrointestinal (N = 222). Whereas patients with neurologic- (N = 391) or nephrologic/hepatologic discharge diagnoses (N = 94) showed no trend.

## Conclusion

Among adult ED patients there is increasing 7-day mortality with increasing lactate level in most patient categories, but patients who are discharged with neurologic- or nephrologic/hepatologic ICD-10 codes have no such trend.

